# Development and Application of Oil-Based Thermo-Responsive Gel Lost Circulation Material SBR−g−PSA

**DOI:** 10.3390/gels12090770

**Published:** 2026-08-27

**Authors:** Zhimin Liu, Chenxi Nie, Fengfeng Xiao, Mengyuan Chen, Ming Liu, Qi Feng, Changtao Yue

**Affiliations:** 1 Drilling Fluid Technology Service Company, Chuanqing Drilling Engineering Company of China National Petroleum Corporation (CNPC), Chengdu 610066, China; liuzm1_sc@cnpc.com.cn (Z.L.); xiaoff_sc@cnpc.com.cn (F.X.); chenmengyuan_sc@cnpc.com.cn (M.C.); lium01_sc@cnpc.com.cn (M.L.); 2 College of Science, China University of Petroleum (Beijing), Beijing 102249, China; niechenxi18@163.com (C.N.); 18563073299@163.com (Q.F.)

**Keywords:** oil-based drilling fluid, lost circulation control, thermo-responsive gel, physical entanglement, polymer network

## Abstract

Lost circulation of oil-based drilling fluids (OBDFs) in deep, fractured formations remains a critical challenge, primarily due to the poor retention and low pressure-bearing capacity of conventional materials on oil-wet rock surfaces. To bridge this gap, this study reports the development and field application of a novel thermo-responsive gel (SBR−g−PSA). Synthesized by grafting highly lipophilic stearyl acrylate (SA) onto a flexible styrene–butadiene rubber (SBR) backbone, the microgel exploits a UCST-like (Upper Critical Solution Temperature) phase transition behavior. At surface temperatures, rigid styrene microdomains act as physical crosslinks, maintaining the particles in a compact, highly dispersible state. Under elevated downhole temperatures (120–180 °C), these microdomains relax, triggering the massive unfolding and physical entanglement of the octadecyl side chains. This in situ topological evolution generates a resilient three-dimensional network with a high thermal degradation temperature of 346.6 °C. Rheological and macroscopic sealing evaluations demonstrate that the gel effectively adapts to OBDFs, securely sealing 1–1.5 mm fractures and limiting fluid loss to within 5 mL under a 4 MPa differential pressure. Successful field application in the deep well Dashen-X reduced severe fluid leakage from 2.42 m^3^/h to zero. This intelligent thermo-responsive platform offers a robust, high-performance technological solution for precise lost circulation control in deep oil and gas exploration.

## 1. Introduction

During drilling operations in fractured formations, oil-based drilling fluids (OBDFs) are highly susceptible to inducing frequent lost circulation [1,2]. The primary reason is that the fracture extension pressure and reopening pressure of OBDFs are lower than those of water-based drilling fluids [3,4,5]. This disparity prevents effective isolation of wellbore pressure, allowing it to continuously act on the fracture tips, thereby exacerbating the formation’s “wedge effect” and promoting further fracture propagation [6,7]. Furthermore, conventional lost circulation materials (LCMs) used in the field (e.g., ultrafine calcium carbonate, mica flakes, and synthetic resins) predominantly exhibit hydrophilic characteristics [8]. This results in poor compatibility with oil-phase media, leading to uneven dispersion and low pressure-bearing strength in OBDFs, rendering them highly vulnerable to erosion and destabilization by fluid flow [9]. Even when certain lipophilic materials are introduced, the oil-wet nature of the wellbore significantly reduces the friction coefficient between the materials and the fracture walls [10]. Consequently, the retention capacity is weakened, making it difficult to form a dense and stable sealing layer, leading to suboptimal overall plugging performance. Therefore, there is an urgent need to develop novel LCMs that possess excellent dispersibility in OBDFs, strong affinity for oil-wet fracture walls, and the ability to rapidly construct high-strength sealing layers downhole, thereby achieving precise, efficient, and long-lasting plugging of oil-phase leakage channels [11,12].

In this context, oil-based gel plugging agents have garnered widespread attention as intelligent plugging systems constructed based on non-covalent interactions [13,14]. These materials primarily rely on intermolecular forces such as hydrogen bonding, van der Waals forces, and hydrophobic associations to self-assemble into three-dimensional network structures within oil-phase environments, facilitating the physical plugging of leakage channels [15,16,17]. Recent advances in polymer chemistry have introduced various low-molecular-weight gelators as potential smart plugging agents. However, these systems fundamentally struggle with an inherent trade-off between thermal stability and gelation speed. They are prone to structural collapse and thermal degradation under the extreme high-temperature and high-differential-pressure conditions of deep wells [18,19]. Furthermore, conventional dynamic non-covalent networks lack robust interfacial adhesion to oil-wet fracture surfaces, leading to seal failure under dynamic fluid washing [20,21]. Therefore, a critical knowledge gap remains: how to design a macromolecular architecture that maintains low-viscosity pumpability at surface conditions, yet autonomously constructs an ultra-strong, thermally resilient three-dimensional sealing network in situ upon high-temperature stimulation.

To overcome these technical challenges, this study utilized macromolecular structural design principles to construct a novel oil-based thermo-responsive gel (SBR−g−PSA), using styrene–butadiene rubber (SBR) as a flexible backbone grafted with a highly lipophilic long-chain monomer, stearyl acrylate (SA) [22,23]. This system utilizes the physical crosslinking of rigid styrene microdomains in the SBR main chain alongside the high lipophilicity of the octadecyl long side chains, endowing the material with specific temperature-responsive characteristics. At ambient temperatures, it disperses stably in OBDFs. As the downhole temperature rises, the intermolecular physical crosslinking nodes gradually activate and loosen, enabling the high-density lipophilic long side chains to fully extend and generate strong physical entanglements in the oil phase [24]. This rapidly induces oil-absorption expansion, constructing a dense and elastic three-dimensional network plug with a pressure-bearing capacity of ≥4 MPa in situ within the 120–180 °C range [25]. This LCM effectively resolves the issues of poor compatibility and high micro-fracture filtration associated with traditional materials, while concurrently overcoming the bottlenecks of poor retention, slow gelation, and insufficient high-temperature strength inherent in existing gels. Ultimately, it provides an effective solution for lost circulation in complex formations, offering robust assurance for safe and efficient deep oil and gas exploration.

## 2. Results and Discussion

### 2.1. Molecular Design and Structural Confirmation

#### 2.1.1. Molecular Design Strategy of SBR−g−PSA

**Figure 1:**
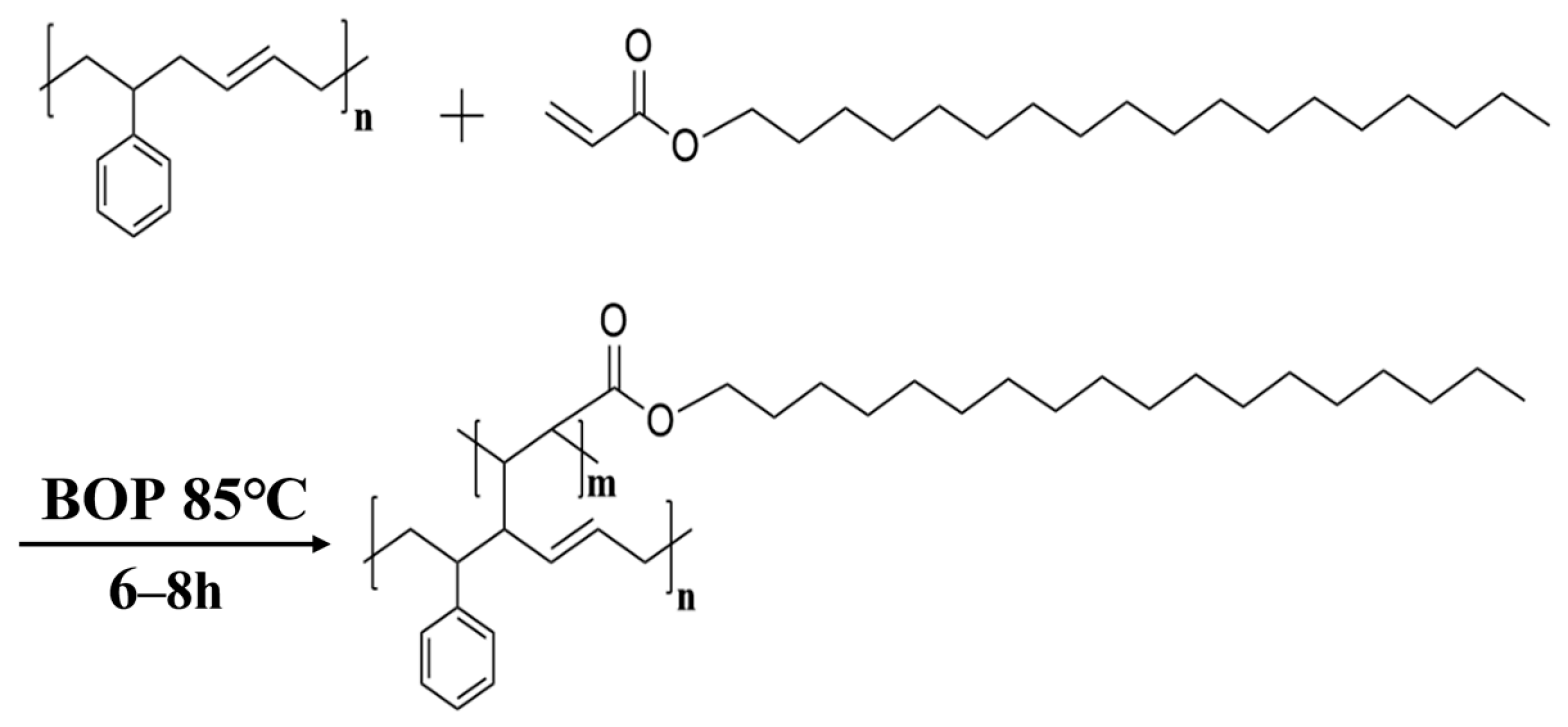
Synthetic route of the SBR−g−PSA graft copolymer.

The molecular architecture of SBR−g−PSA was designed to bridge the gap between surface pumpability and downhole sealing integrity in OBDFs. Styrene–butadiene rubber (SBR) was selected as the flexible polymer backbone due to its excellent oil compatibility and the presence of rigid styrene microdomains, which act as robust physical crosslinking nodes at lower temperatures [26]. To impart strong affinity for the oil phase and trigger temperature-responsive expansion, stearyl acrylate (SA), a monomer featuring a long octadecyl carbon chain (C_18_), was grafted onto the SBR backbone [27]. The overall synthetic route for this graft copolymer is illustrated in Figure 1.

This specific combination creates a thermo-responsive mechanism: at surface and shallow wellbore temperatures, the physical crosslinking limits the extension of the grafted side chains, maintaining the microgel as compact particles that facilitate easy pumping and minimal impact on drilling fluid rheology. However, upon reaching the high-temperature loss zones, the thermal energy disrupts the glassy state of the styrene microdomains, loosening the physical constraints. Consequently, the high-density, highly lipophilic octadecyl side chains spontaneously unfold into the oil-continuous phase. The subsequent intense hydrophobic associations and physical entanglements among these extended chains drive massive oil absorption and volumetric swelling, resulting in the in situ formation of a three-dimensional plugging network.

#### 2.1.2. Fourier-Transform Infrared (FTIR) Spectroscopy

To investigate the molecular chain structure of the synthesized product, FTIR spectroscopy was performed on the purified polymer.

**Figure 2:**
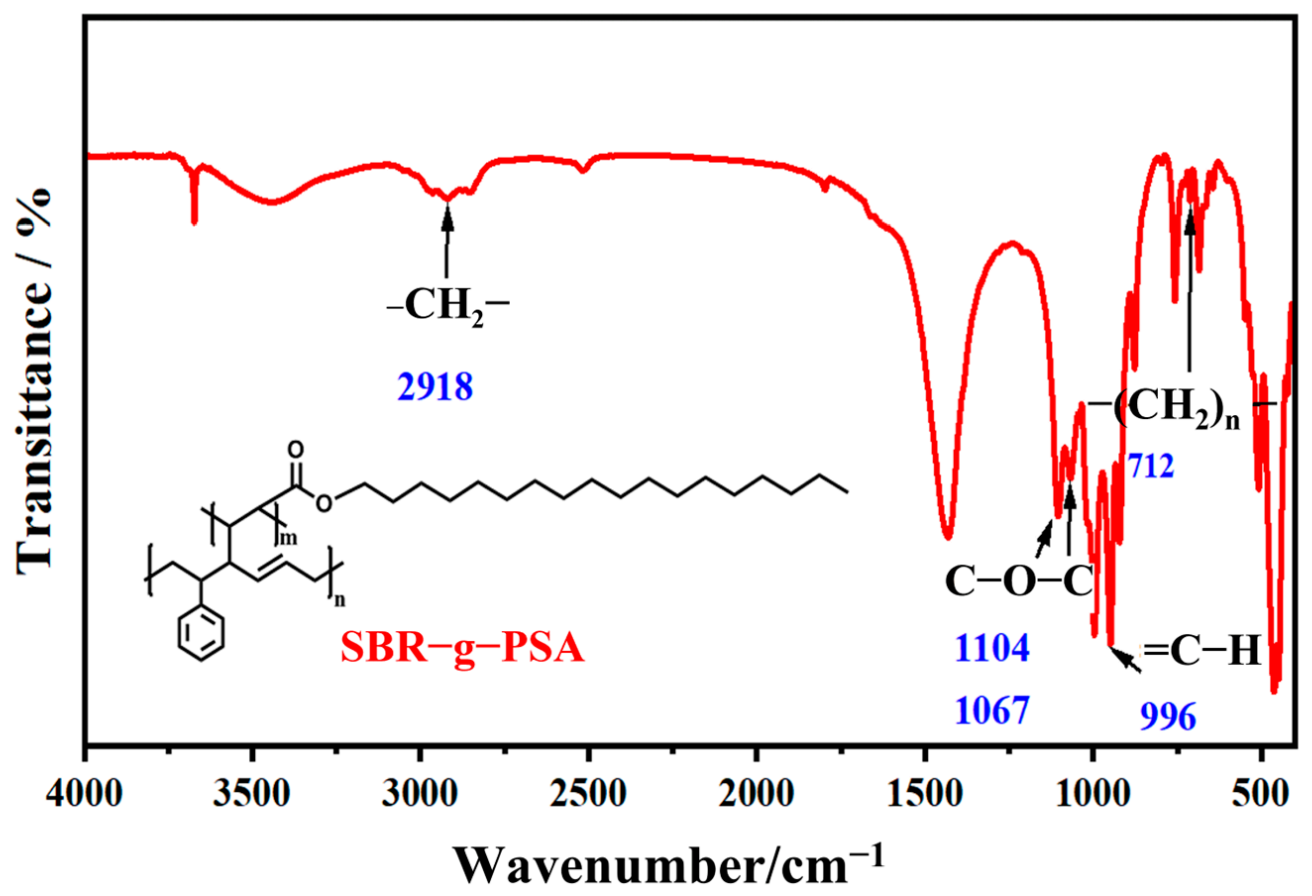
FTIR spectrum of the synthesized SBR−g−PSA.

In the infrared spectrum, a prominent antisymmetric stretching vibration peak of the methylene group (-CH_2_-) in the long alkyl chains appeared at 2918 cm^−1^, indicating the presence of abundant saturated long carbon chains within the system. Concurrently, the asymmetric and symmetric stretching vibration characteristics of the ester group (C-O-C) clearly appeared at 1104 cm^−1^ and 1067 cm^−1^. It is worth noting that the typical carbonyl (C=O) stretching peak around 1730 cm^−1^ is relatively weak, which may be partially attributed to the severe steric shielding provided by the high-density octadecyl chains and the intense dipole–dipole interactions within the compact, tightly coiled polymer network in its dry, low-temperature state, which significantly restricts the stretching vibration of the C=O bonds. Nevertheless, the prominent C-O-C peaks, combined with the characteristic in-plane rocking vibration of long-chain methylenes at 712 cm^−1^, provide conclusive evidence that the highly non-polar octadecyl ester groups were successfully incorporated into the macromolecular network [28]. Additionally, the strong absorption peak at 1431 cm^−1^ is attributed to the overlapping absorption of the scissoring bending vibration of methylene groups on the alkyl chains and the C=C stretching vibration of the benzene ring skeleton; the characteristic peak at 996 cm^−1^ corresponds to the out-of-plane bending vibration of =C-H in the trans-1,4-structure of the butadiene segments, confirming the presence of the polybutadiene backbone and a small number of phenyl groups. Notably, the absorption peak at 712 cm^−1^ holds crucial structural significance: it belongs to the in-plane rocking vibration of methylene groups in long alkyl chains (appearing when *n* ≥ 4 in -(CH_2_)n-), conclusively proving that the highly lipophilic octadecyl long chains with 18 carbon atoms were successfully incorporated into the macromolecular network. In summary, the FTIR analysis clearly demonstrates that the product simultaneously contains olefinic double bonds, benzene rings, and long-chain aliphatic ester groups, preliminarily verifying the successful synthesis of the ternary graft copolymer. The FTIR spectrum of the synthesized product is shown in Figure 2.

#### 2.1.3. Proton Nuclear Magnetic Resonance (^1^H-NMR) Spectroscopy

**Figure 3:**
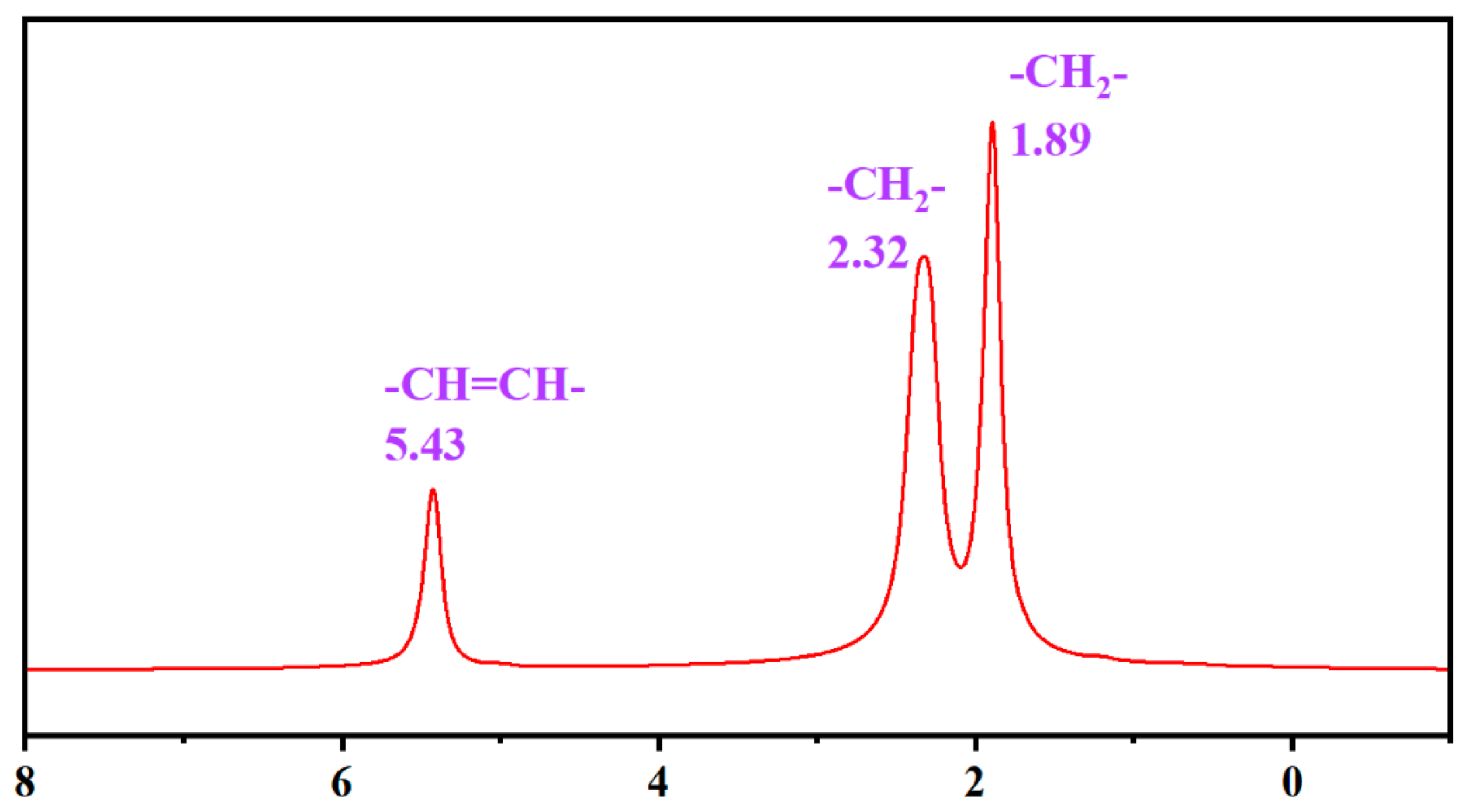
Proton nuclear magnetic resonance (^1^H-NMR) spectrum of SBR−g−PSA.

Building upon the chemical composition confirmed by FTIR, solid-state proton nuclear magnetic resonance (solid-state ^1^H-NMR) spectroscopy further elucidated the aggregation morphology and segmental motion characteristics of the graft copolymer. The ^1^H−NMR spectrum of SBR−g−PSA is presented in Figure 3.

In the solid-state NMR spectrum, only the broad olefinic proton (-CH=CH-) peak at 5.43 ppm and the extremely strong aliphatic methylene proton (-CH_2_-) peaks at 2.32 ppm and 1.89 ppm were clearly observed. These signals are primarily attributed to the highly flexible and mobile polymer backbone segments and the long alkyl side chains. By integrating the main peaks in the spectrum, it was found that the integration area of the saturated aliphatic hydrogens at 1.89 ppm and 2.32 ppm is much larger than that of the double-bond hydrogens at 5.43 ppm. This phenomenon corroborates the strong long-chain alkyl peaks observed in the FTIR data, providing robust evidence from a macromolecular perspective that a high density of octadecyl long chains was successfully grafted onto the polymer backbone. Concurrently, the aromatic proton signals corresponding to the styrene microdomains (typically around 6.5–7.5 ppm) are entirely absent from the spectrum. This is fundamentally explained by the solid-like state and the varying spin–spin relaxation times (T_2_) of different segments within the gel network [29]. The rigid styrene microdomains act as physical crosslinking nodes; their localized motion is extremely restricted, resulting in a very short T_2_ relaxation time. Consequently, their NMR signals broaden excessively and merge into the baseline. In contrast, the highly mobile polybutadiene backbone and the flexible octadecyl side chains maintain longer T_2_ times, yielding the sharp peaks observed at 5.43 ppm, 2.32 ppm, and 1.89 ppm. This distinct contrast in segmental mobility confirms the heterogeneous network topology of SBR−g−PSA, consisting of rigid crosslinking cores and highly flexible side chains. The classification of SBR−g−PSA as a physical gel is fundamentally grounded in its non-covalent network architecture. Unlike conventional chemically crosslinked networks, the three-dimensional matrix in SBR−g−PSA is sustained by reversible physical associations, including long-chain hydrophobic interactions among octadecyl (C18) groups and van der Waals forces. This transient physical crosslinking mechanism is macroscopically corroborated by its dynamic viscoelastic performance (G′, G″, tanδ < 0.15), confirming the formation of a physical network.

#### 2.1.4. Micro-Morphology Analysis

**Figure 4:**
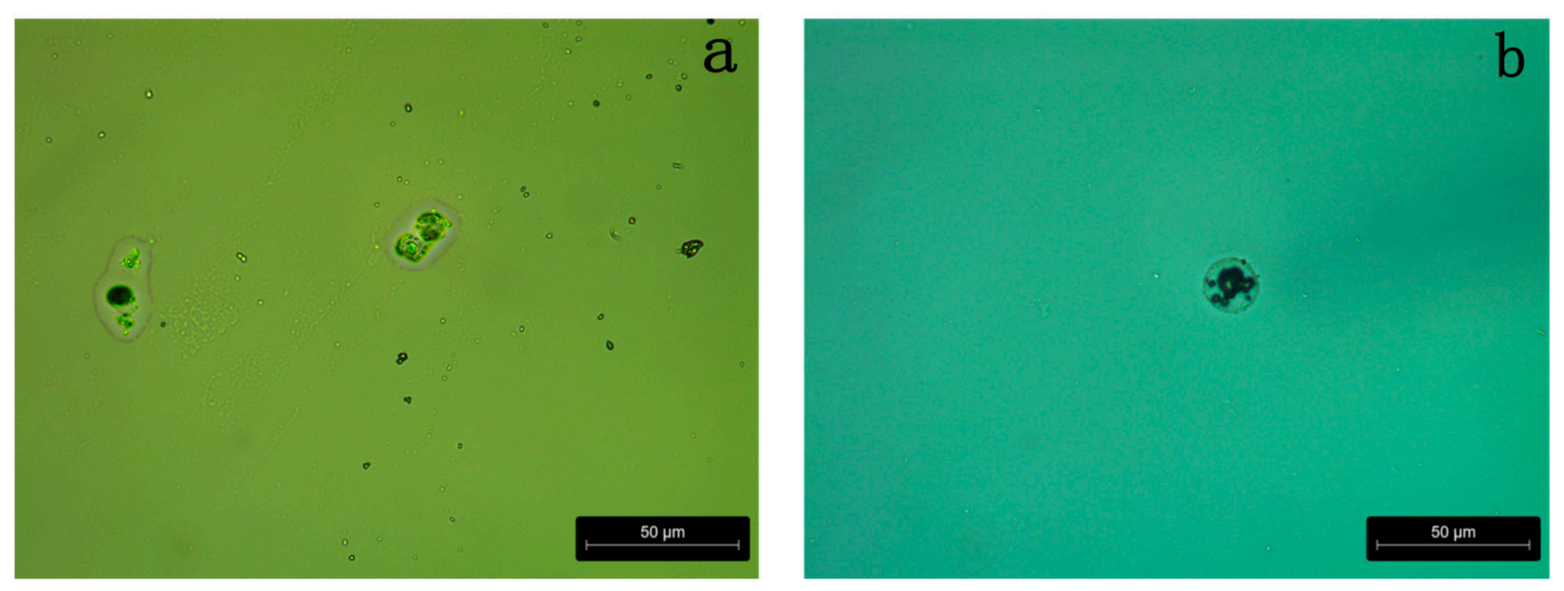
Optical microscopy images of SBR−g−PSA: (**a**) uniformly sized spherical particles with smooth surfaces; (**b**) irregular assemblies formed by the aggregation of multiple small particles.

The micro-morphology of the samples was investigated using optical microscopy. The micro-morphology of SBR−g−PSA was examined by optical microscopy, as shown in Figure 4.

SBR−g−PSA exhibits two typical microscopic morphologies: one consists of uniformly sized spherical particles with smooth surfaces and excellent dispersibility, showing no obvious aggregation; the other consists of irregular assemblies formed by the aggregation of multiple small spherical particles. Further observation revealed that all assembled structures display a distinct microphase aggregation pattern. Based on the molecular design, it can be inferred that the lipophilic components likely act as the aggregation core, providing oil-phase compatibility, while the flexible polybutadiene main chain segments entangle around the exterior of the crosslinked core, forming a network skeleton. This specialized structural distribution effectively balances the gel’s dispersibility and oil-absorbing swelling capability in OBDFs, confirming that the material’s micro-morphology is entirely consistent with the expected molecular design.

#### 2.1.5. Particle Size Analysis

**Figure 5:**
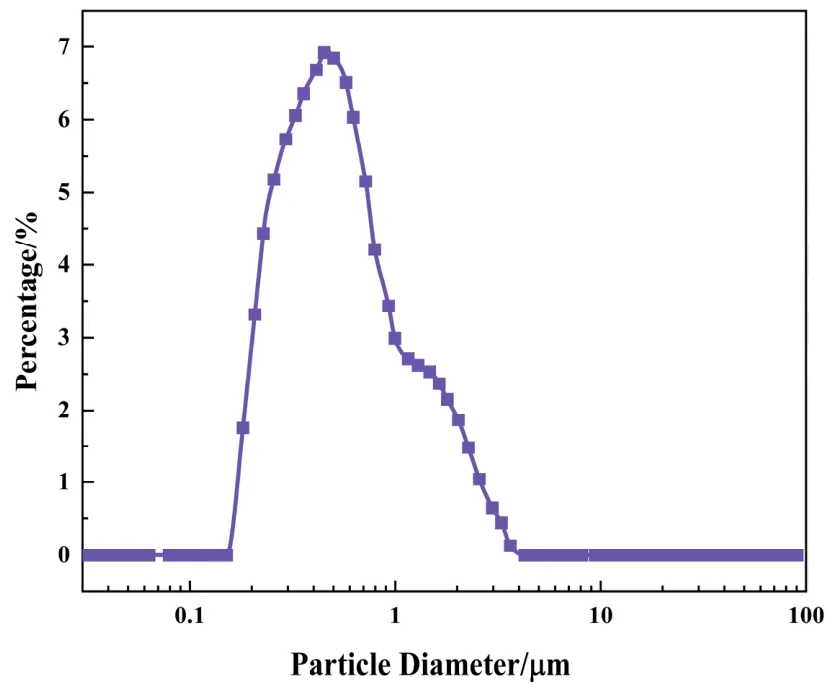
Particle size distribution curve of the SBR−g−PSA microgel.

Particle size distribution was analyzed using a laser diffraction particle size analyzer. The particle size distribution of SBR−g−PSA microgel is presented in Figure 5.

Particle size distribution of SBR−g−PSA microgel was characterized using a laser diffraction particle size analyzer. The obtained particle size distribution presents a narrow and nearly symmetric profile without obvious impurity-related secondary peaks. The characteristic particle size parameters directly output by the instrument are D_10_ = 0.21 μm, D_50_ = 0.36 μm, and D_90_ = 0.88 μm. The median particle diameter D_50_ is close to the theoretical prediction range of 0.48–0.52 μm derived from monomer feeding ratio and polymerization conditions. The micro–nano-sized initial particles of SBR−g−PSA are capable of penetrating into micro-scale pore throats. Benefiting from the oil-absorbing swelling performance under high-temperature reservoir conditions, the material can further seal millimeter-scale fracture apertures. Such multi-scale size adaptability covering nano-, micro-, and millimeter scales lays a physical foundation for its full-scale plugging performance. These results verify the reliability of the material preparation route and demonstrate that the proposed molecular design strategy can effectively regulate the particle dimension of the as-synthesized gel, which provides favorable prerequisites for plugging leakage channels with diverse sizes in oil–gas formations.

#### 2.1.6. Thermal Stability

**Figure 6:**
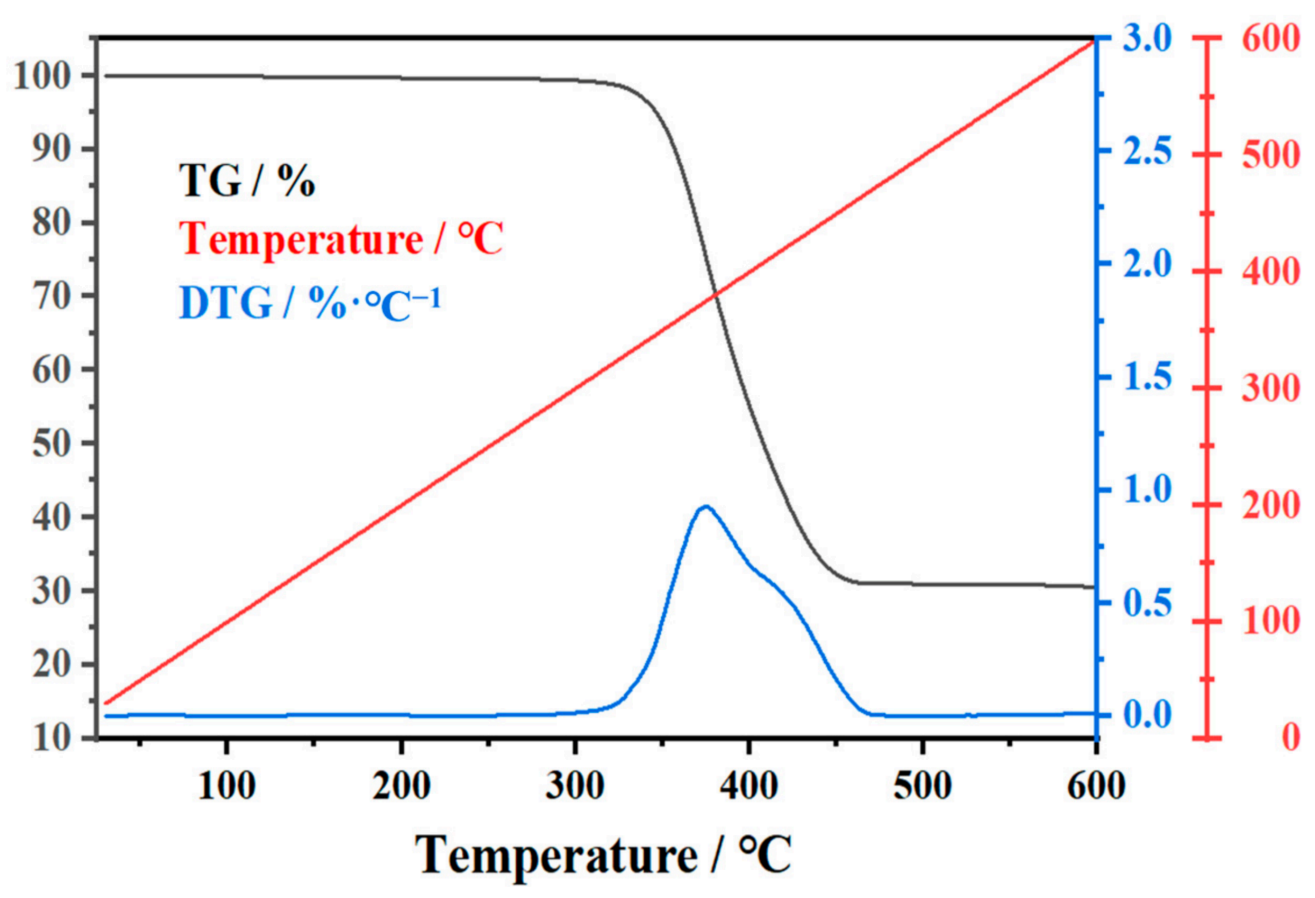
Thermogravimetric analysis (TGA) and derivative thermogravimetry (DTG) curves of SB−g−PSA.

The TGA and DTG curves of SBR−g−PSA are shown in Figure 6. Thermogravimetric analysis (TGA) was utilized to evaluate the thermal stability of the oil-based thermo-responsive gel.

The thermal stability and degradation characteristics of the gel were evaluated via TGA. The samples were heated up to 600 °C, and the resulting TGA and derivative thermogravimetry (DTG) curves were recorded to determine the onset thermal degradation temperature (T5%), maximum thermal decomposition rate temperature (Tmax), and final char yield. In the TGA and derivative thermogravimetry (DTG) curves, the material demonstrated stable thermal behavior, with an onset thermal degradation temperature (T_5%_, representing 5% weight loss) as high as 346.6 °C. This extremely high thermal resistance threshold far exceeds the conventional high-temperature operational environments (e.g., 140–160 °C) encountered by deep shale gas drilling fluids, fully proving the high thermodynamic stability of the ternary graft copolymer network containing physical crosslinking points. As the temperature continued to increase, the DTG curve indicated that the maximum thermal decomposition rate temperature (T_max_) occurred at 374.5 °C. This severe weight loss stage is primarily attributed to the scission of the SBR−g−PSA organic polymer backbone and the thermal degradation and volatilization of the abundant octadecyl side chains [30]. Notably, when the test temperature reached 600 °C, the polymer matrix had essentially decomposed, but the TGA curve eventually plateaued, retaining a high solid residue of 30.54%. Since pure organic polymers typically undergo massive decomposition at this temperature, this high residual mass of 30.54% is primarily due to complex crosslinking and carbonization reactions of the polymer backbone (especially the double bond-containing polybutadiene segments) during high-temperature thermal degradation, forming a stable residual carbon skeleton. This relatively high char yield further corroborates the excellent structural stability of the SBR−g−PSA crosslinked network under extreme high-temperature conditions.

### 2.2. Performance Evaluation

#### 2.2.1. Rheological Enhancement in Drilling Fluids

**Figure 7:**
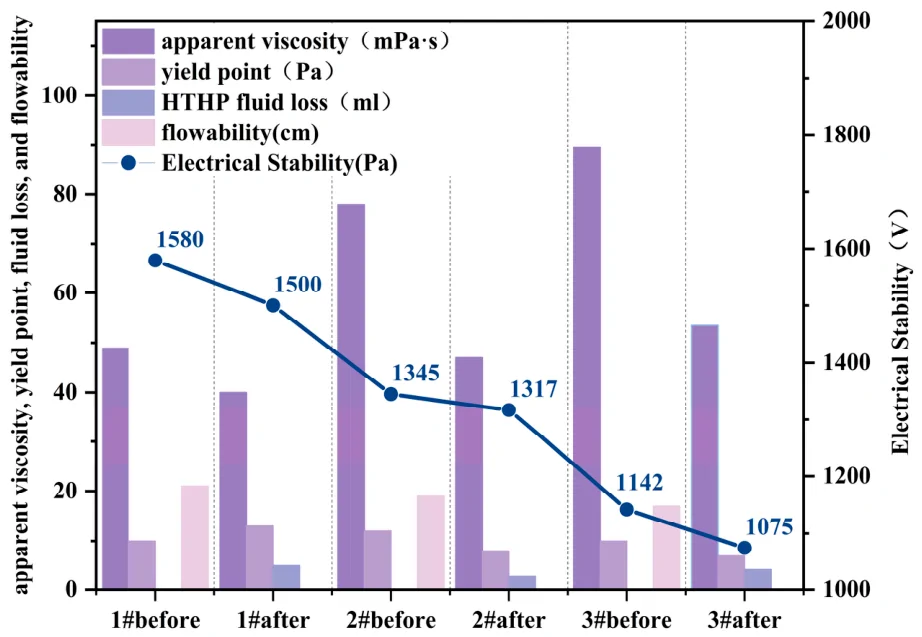
Comparison of the rheological properties and electrical stability of drilling fluids before and after aging.

The rheological properties and electrical stability of the drilling fluids before and after aging are compared in Figure 7. To systematically evaluate the performance of SBR−g−PSA under practical wellbore conditions, its influence on the rheological behavior and electrical stability of standard oil-based drilling fluids was investigated before and after thermal aging. In this study, a standard OBDF formulation (1#) was used as the base fluid. SBR−g−PSA was added at 5% (2#) and 10% (3#) mass fractions, and the rheological properties were evaluated before and after aging (16 h at 160 °C).

Before aging, the apparent viscosity (AV) of the 1# base fluid was 49 mPa·s, which increased to 53.5 mPa·s in the 3# system. This moderate thickening effect indicates that SBR−g−PSA can effectively enhance the cutting-carrying capacity of the system, while the 53.5 mPa·s value remains within the safe circulation viscosity range for field operations (≤50–60 mPa·s) [31]. The yield point (YP) slightly increased with the addition of SBR−g−PSA, which is advantageous for maintaining wellbore stability. High-temperature, high-pressure (HTHP) fluid loss tests demonstrated that the aged 1# base fluid had a fluid loss of 5.0 mL, whereas the aged 3# system experienced a significant reduction to 4.2 mL. This indicates that SBR−g−PSA can form a dense three-dimensional network on the filter cake surface, effectively blocking filtrate invasion into the formation [32]. The electrical stability (ES) dropped from 1580 V (1# before aging) to 1075 V (3# after aging) but still remained well above the industry minimum safety threshold (≥500 V) [33], demonstrating that the emulsion system maintains robust resistance to demulsification [34]. The stability of the yield point and other rheological parameters confirms that the flow behavior is smooth. Overall, SBR−g−PSA increases viscosity and reduces fluid loss while preserving excellent rheological and electrochemical stability, thereby comprehensively enhancing the overall performance of the drilling fluid. Notably, because the continuous phase of the testing fluid contained a 25 wt% NaCl brine phase alongside emulsifiers and solid weighting agents, these stable rheological and filtration results demonstrate that the polymer network possesses strong tolerance against high salinity and solid-phase interference during drilling operations.

#### 2.2.2. Gelation Time

**Figure 8:**
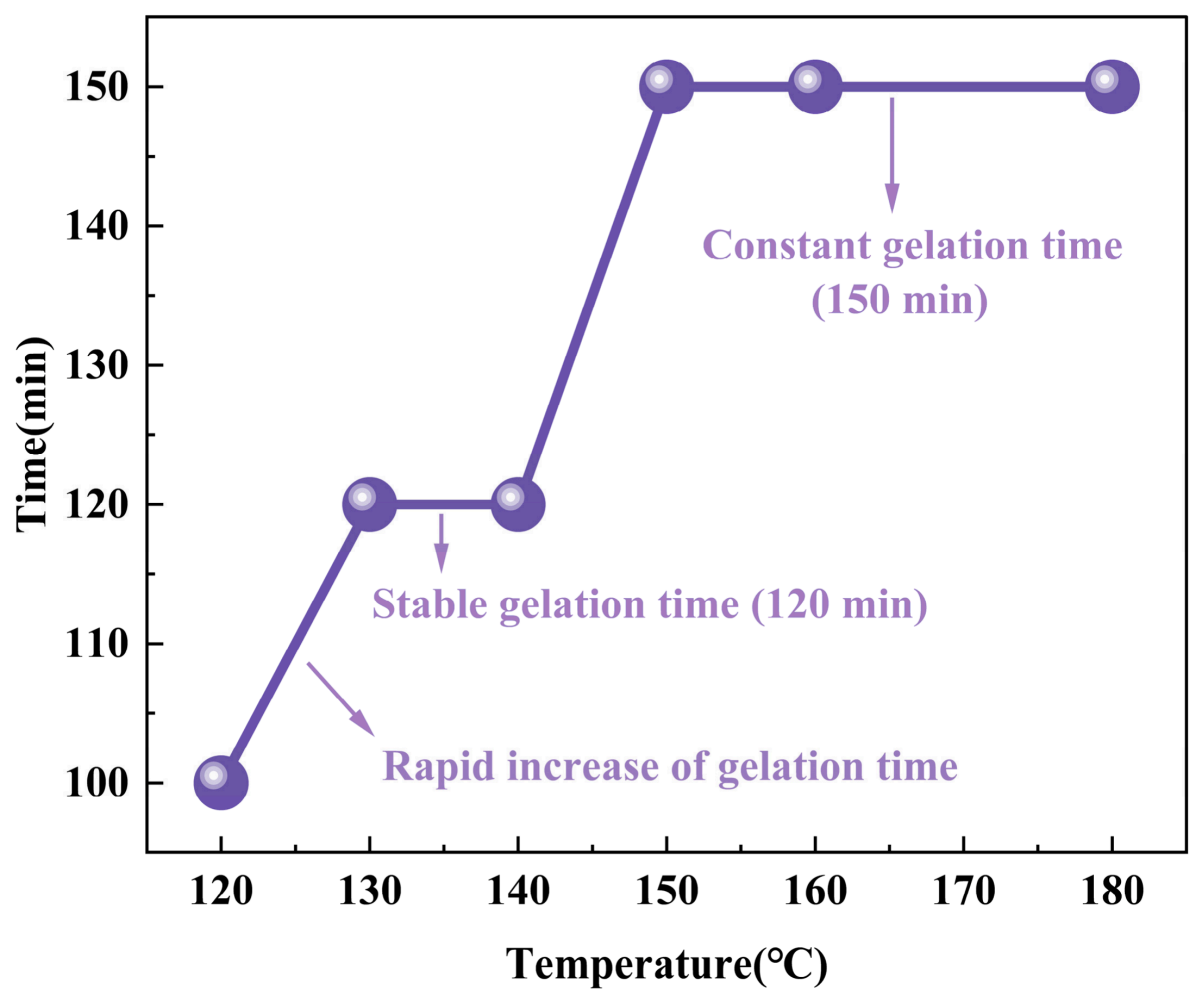
Gelation time of SBR−g−PSA as a function of temperature.

At 120 °C, the gelation time is 100 min; as the temperature rises to 130 °C and 140 °C, the gelation time extends to 120 min; and between 150 °C and 180 °C, the gelation time remains stable at 150 min. As discussed in Section 2.3.2, temperatures above 150 °C cause intense thermal motion of octadecyl side chains, which continuously breaks interchain physical entanglements. A fixed 150 min is required to reach dynamic balance between entanglement assembly and thermal dissociation, so gelation time no longer rises within 150–180 °C. These results demonstrate that the gelation time of SBR−g−PSA exhibits regular and stable variation characteristics within the 120–180 °C range, showing excellent gelation time controllability [35]. A stable gelation time allows field operators to better control pumping schedules and plugging quality, fully satisfying the requirements for safe eld operations, as illustrated in Figure 8.

#### 2.2.3. Adhesion and Retention Capabilities

**Figure 9:**
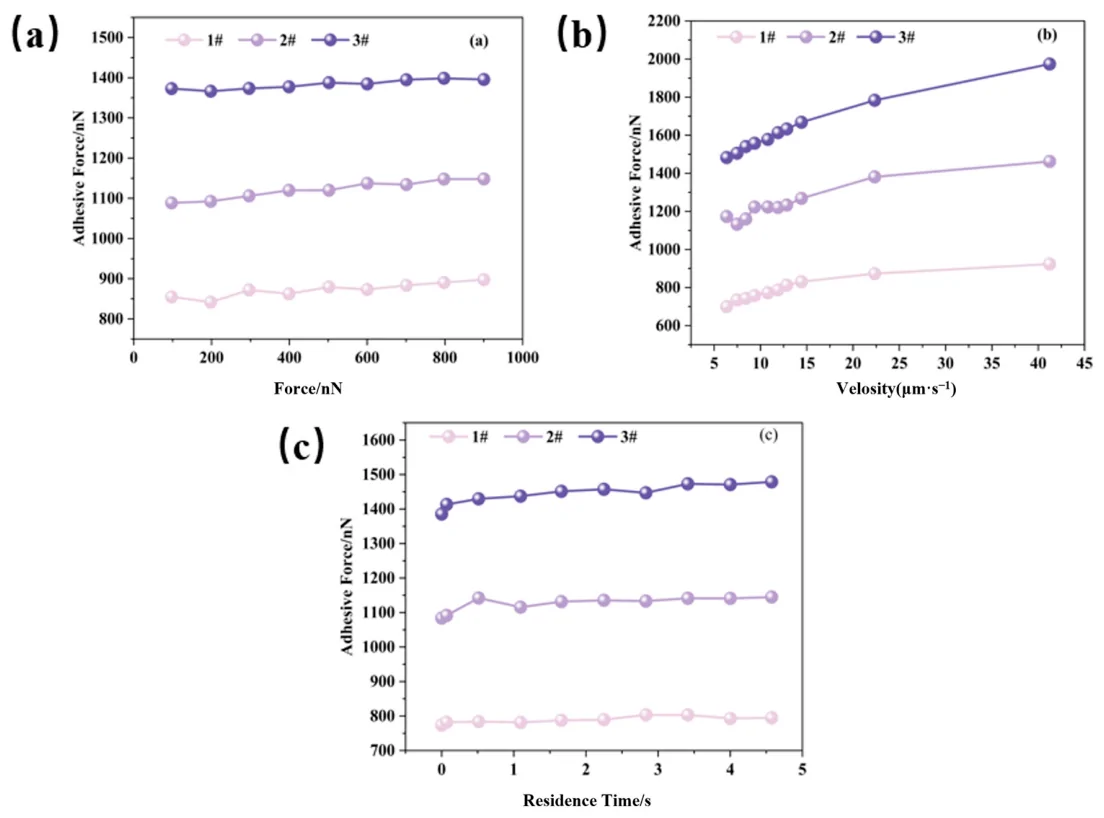
Effects of (**a**) applied force, (**b**) penetration speed, and (**c**) residence time on the atomic force adhesion of drilling fluids with varying SBR−g−PSA concentrations.

The effects of applied force, penetration speed, and residence time on adhesion are shown in Figure 9. To elucidate the mechanism by which SBR−g−PSA enhances drilling fluid stability, micro-mechanical evaluations were conducted focusing on the applied force of the probe, the penetration speed of the probe, and the probe’s residence time on the sample surface. Using the standard OBDF (1#) as a control, the atomic force adhesion between the SiO_2_ microsphere probe and drilling fluids containing 5% (2#) and 10% (3#) SBR−g−PSA was tested [36]. This value directly evaluates the effect of SBR−g−PSA on improving the retention performance of the drilling fluid on rock surfaces (quartz sand).

The 1# fluid exhibited the lowest adhesive force, while the 3# system exhibited the highest. The overall trend indicates that higher SBR−g−PSA concentrations lead to stronger adhesion between the drilling fluid and quartz sand. At a constant SBR−g−PSA concentration, the adhesive force between the probe and the drilling fluid gradually increased as the probe’s applied force increased from 100 nN to 1000 nN, as the residence time extended from 0.1 s to 5.0 s, and as the probe speed increased from 3 μm/s to 78.1 μm/s. This is because, at higher relative movement speeds, the dynamic entanglement and frictional interactions between the polymer chain segments in the gel system and the oil-phase molecules are significantly enhanced, rapidly elevating the adhesive resistance [37]. This property demonstrates that SBR−g−PSA significantly enhances the microscopic interfacial friction and adhesion between the plugging material and the rock matrix, thereby improving its resistance to dynamic fluid washout and retention capabilities within leakage formations.

#### 2.2.4. Dynamic Viscoelasticity and Gel Strength

**Figure 10:**
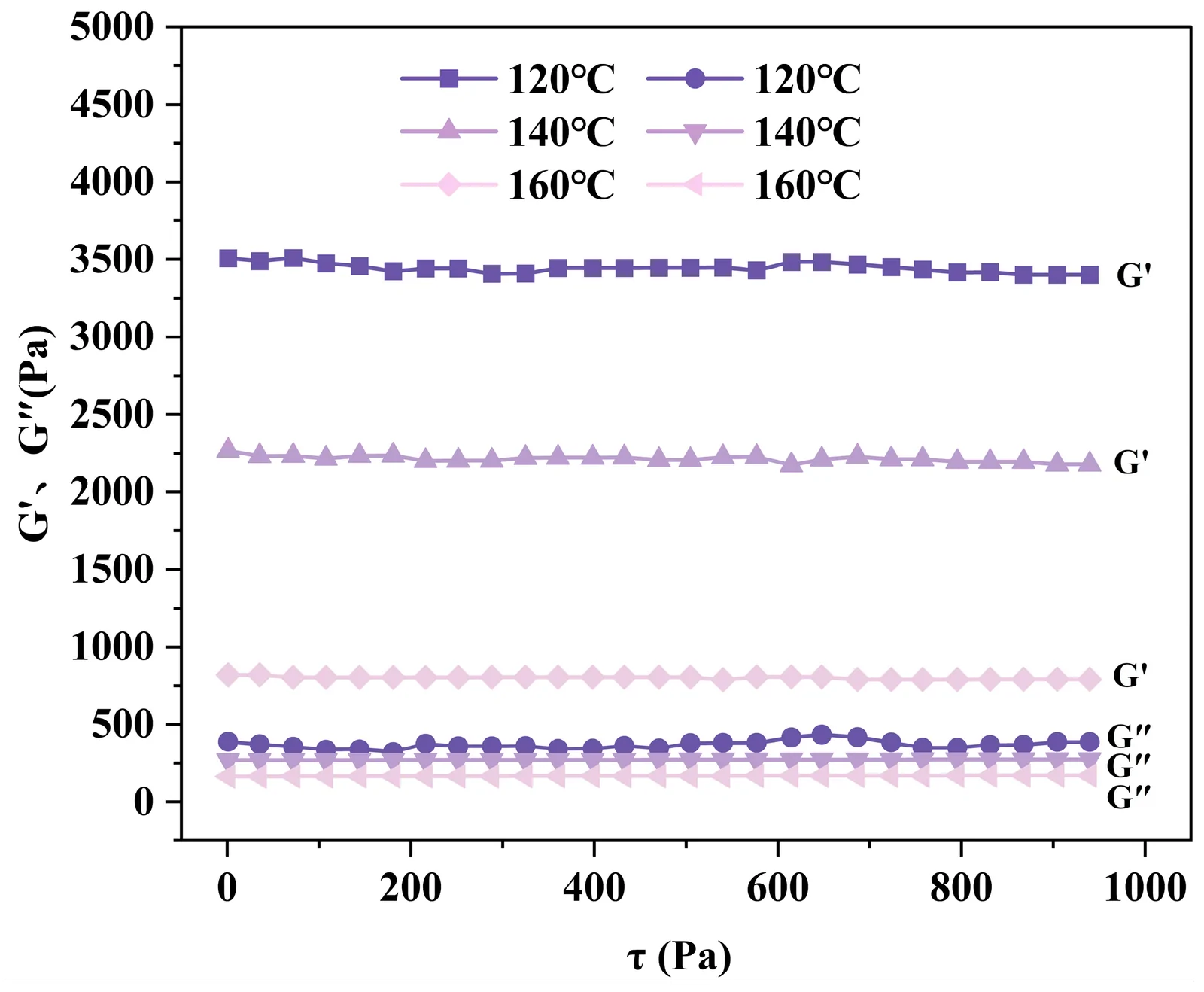
Oscillatory stress sweep moduli (G′ and G″) of SBR−g−PSA after 72 h of aging at different high temperatures.

The oscillatory stress sweep moduli of SBR−g−PSA after aging at different temper-atures are presented in Figure 10. The mechanical strength of the oil-based thermo-responsive gel was evaluated using dynamic oscillatory shear sweeps after 72 h of high-temperature aging at 120 °C, 140 °C, and 160 °C.

The results indicate that throughout the entire stress sweep range, G′ sequentially stabilized at approximately 5000 Pa, 3500 Pa, and 2000 Pa, consistently remaining much higher than the corresponding G″ values (approximately 300 Pa, 200 Pa, and 100 Pa). The loss factor (tan δ) remained below 0.15, displaying typical solid-like elastic characteristics [38]. Furthermore, the modulus plateau did not experience a sharp decline as shear stress increased, demonstrating that the network structure was not substantially disrupted under strong shear [39]. Although G′ experienced a moderate decrease from approximately 5000 Pa to 2000 Pa as the aging temperature increased from 120 °C to 160 °C—which can be attributed to the intensified thermal motion of polymer segments partially disrupting the physical entanglements—the consistent dominance of G′ over G″ (with tan δ remaining below 0.15) confirms that the gel retains a stable, solid-like elastic network. This residual mechanical strength proves that SBR−g−PSA possesses sufficient thermodynamic stability and resilient elasticity under severe conditions up to 160 °C, fully satisfying the pressure-bearing requirements of deep-well high-temperature plugging operations.

#### 2.2.5. Fracture Sealing Performance

**Figure 11:**
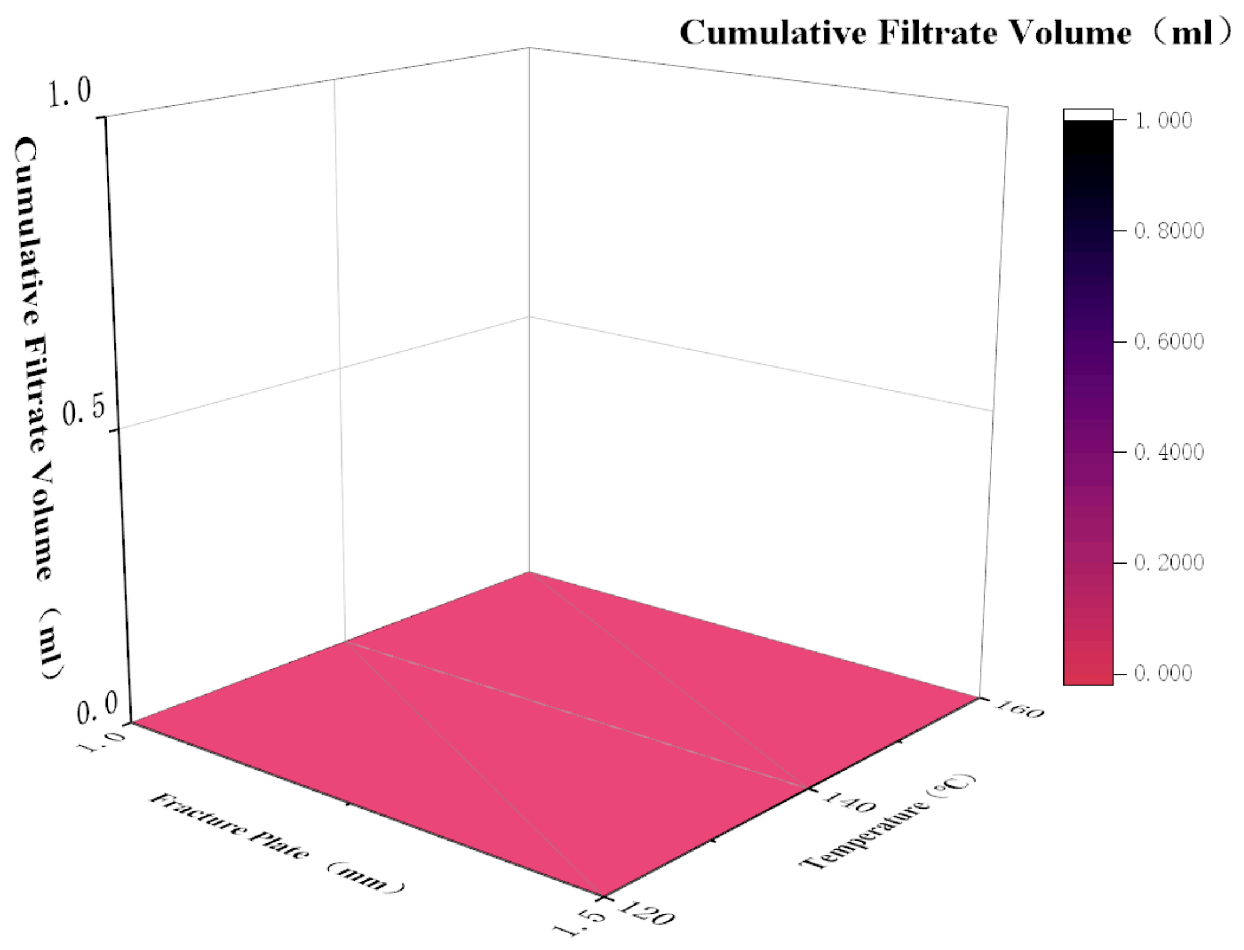
Dynamic sealing performance of the SBR−g−PSA system in 1 mm and 1.5 mm slotted plate tests.

The dynamic sealing performance of the SBR−g−PSA system in slotted plate tests is shown in Figure 11. A high-temperature–high-pressure (HTHP) lost circulation tester was utilized to conduct fracture sealing tests in this study. Diesel was used as the base fluid, with a 10% mass fraction of SBR−g−PSA added as the plugging material. Standard metal slotted plates of 1 mm and 1.5 mm were used to simulate formation fractures.

The test results demonstrated the sealing capacity of SBR−g−PSA under HTHP conditions. Instead of acting as traditional discrete bridging particles, the submicron SBR−g−PSA particles underwent massive thermal-induced expansion and interchain physical entanglement at elevated temperatures. These particles fused into a continuous, macroscopic high-strength gel plug that completely filled the 1–1.5 mm slots. The results demonstrate that in the temperature range of 120–160 °C, no fluid leakage was observed through the 1 mm and 1.5 mm slotted plates under any of the tested pressure differentials (0–4 MPa). This provides direct confirmation that the sealing layer constructed by the gel possesses exceptional pressure-bearing capacity, capable of withstanding pressure differentials up to 4 MPa without rupturing. This indicates that SBR−g−PSA not only features excellent temperature responsiveness but also establishes a stable and reliable sealing barrier against fracture-induced leakage in high-temperature environments, serving as a highly effective solution for lost circulation in deep, high-temperature fractured wells [40].

#### 2.2.6. Sand Bed Plugging Performance

**Figure 12:**
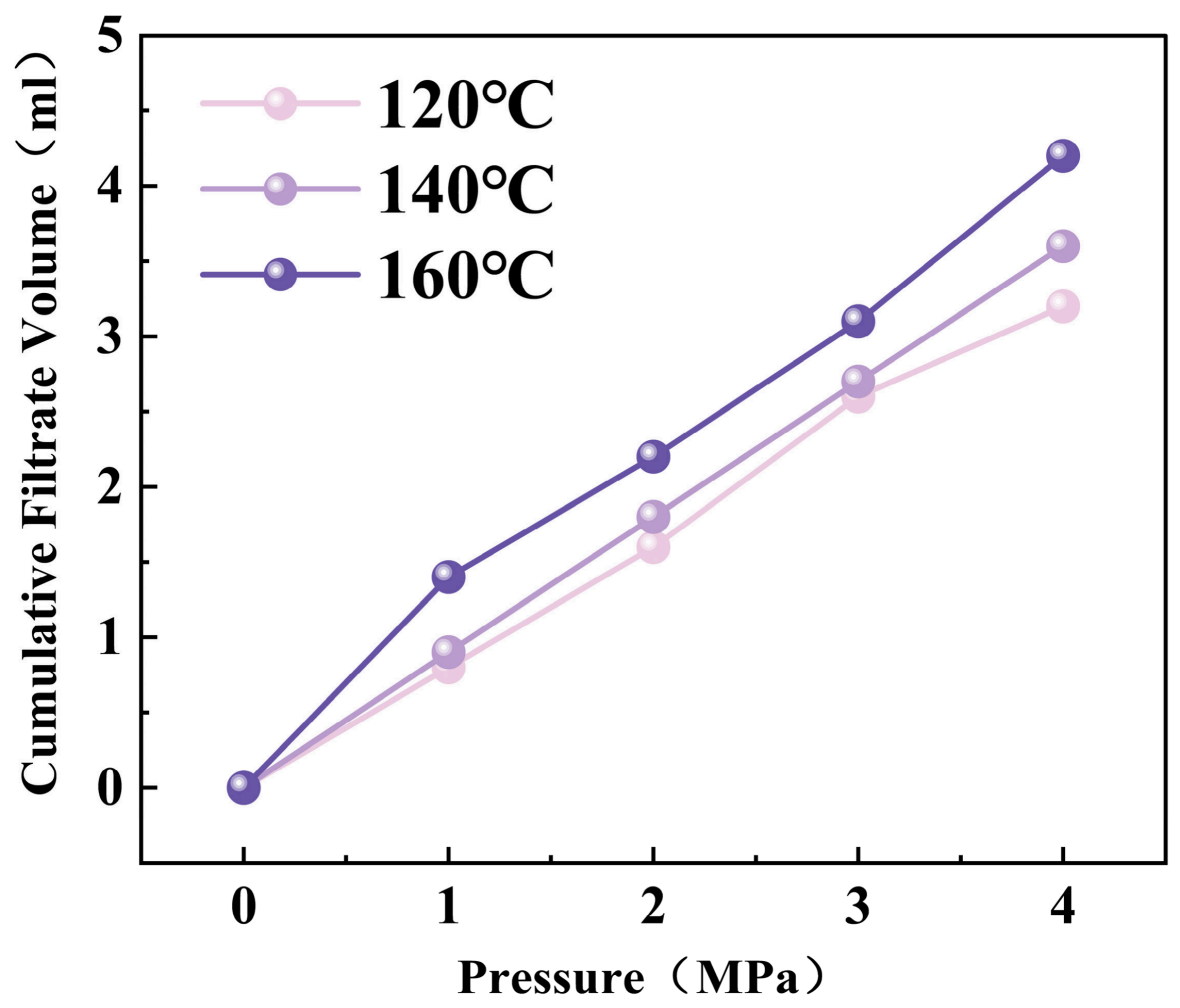
Cumulative fluid loss of the SBR−g−PSA system in 20–40 mesh sand bed plugging tests at various temperatures.

The cumulative fluid loss of the SBR−g−PSA system in sand bed plugging tests is presented in Figure 12. The plugging performance of SBR−g−PSA in permeable formations was further evaluated via sand bed sealing tests using an HTHP lost circulation tester.

The prepared plugging fluid (diesel-based OBDF + 10% SBR−g−PSA) was poured into the tester containing 20–40 mesh quartz sand. The system was pressurized, heated, and stabilized according to standard procedures, with cumulative fluid loss recorded [41]. SBR−g−PSA demonstrated excellent initial sealing capabilities at all three test temperatures (120 °C, 140 °C, and 160 °C), exhibiting 0 mL fluid loss at 0 MPa. As the test temperature increased, the compressive resistance of the gel experienced a minor, controllable decline: under the same test pressure, cumulative fluid loss slightly increased. At a 4 MPa differential pressure, the cumulative fluid loss was 3.2 mL at 120 °C, 3.6 mL at 140 °C, and 4.2 mL at 160 °C. This phenomenon is directly related to the temperature dependence of gel strength; elevated temperatures cause a moderate reduction in the macromolecular gel network’s modulus, allowing a marginal amount of filtrate to pass through under high pressure. The experimental results conclusively prove that the gel exhibits significant temperature responsiveness in the 120–160 °C range. Even under the severe combined conditions of 160 °C and 4 MPa, the cumulative fluid loss was strictly constrained to within 5 mL, far surpassing traditional rigid plugging materials and maintaining outstanding overall leak prevention and plugging efficacy.

### 2.3. Mechanism Analysis

#### 2.3.1. Thermo-Responsive Phase Transition Behavior

**Figure 13:**
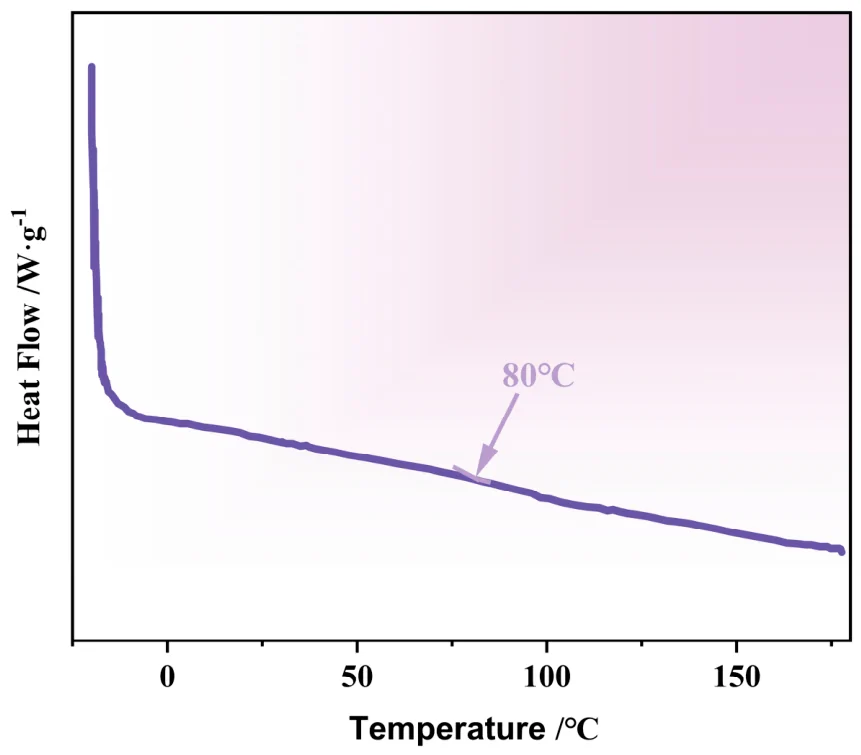
DSC curve of SBR−g−PSA.

The DSC curve of SBR−g−PSA is shown in Figure 13. The unique thermodynamic transition characteristics of SBR−g−PSA dictate its intelligent responsive behavior to varying downhole environmental temperatures.

A distinct endothermic step transition near 80 °C is clearly observable in the DSC heat flow curve. Based on an analysis of the macromolecular network topology of SBR−g−PSA, this transition temperature (Tg ≈ 80 °C) corresponds to the glass transition of the rigid styrene microdomains (PS) within the polymer network [42], or the dissociation and activation temperature of the physical crosslinking nodes.

When the drilling fluid is under low-temperature conditions (e.g., circulating near the wellhead or in shallow sections, T < 80 °C), the rigid microdomains acting as physical crosslinking points are in a glassy state. The motion of the molecular chain segments is severely restricted, and the entire graft copolymer network is highly contracted and “frozen”. At this stage, the material exists as uniformly dispersed micro/nanoscale particles in the OBDF, exerting minimal impact on the overall rheology of the system and ensuring excellent pumpability. However, as the drilling fluid circulates into deep, high-temperature lost circulation zones (T > 80 °C), the rigid crosslinking microdomains absorb heat, activate, and loosen. The molecular chain segments gain sufficient motional freedom, and the oil-phase medium rapidly diffuses and permeates into the polymer network. This triggers significant physical swelling and a macroscopic conformational transition of the graft copolymer. The thermo-responsive behavior of SBR−g−PSA in the continuous oil phase exhibits an upper critical solution temperature (UCST)-like phase transition. Below Tg (~80 °C), the macromolecular network is restricted in an enthalpy-favored collapsed state due to rigid polystyrene domain crosslinking. Above 80 °C, thermal energy induces phase mixing and loosens these domain nodes, enabling the highly lipophilic octadecyl side chains to fully uncoil and solubilize in the oil matrix—a process driven by the gain in conformational entropy.

#### 2.3.2. Plugging Mechanism

**Figure 14:**
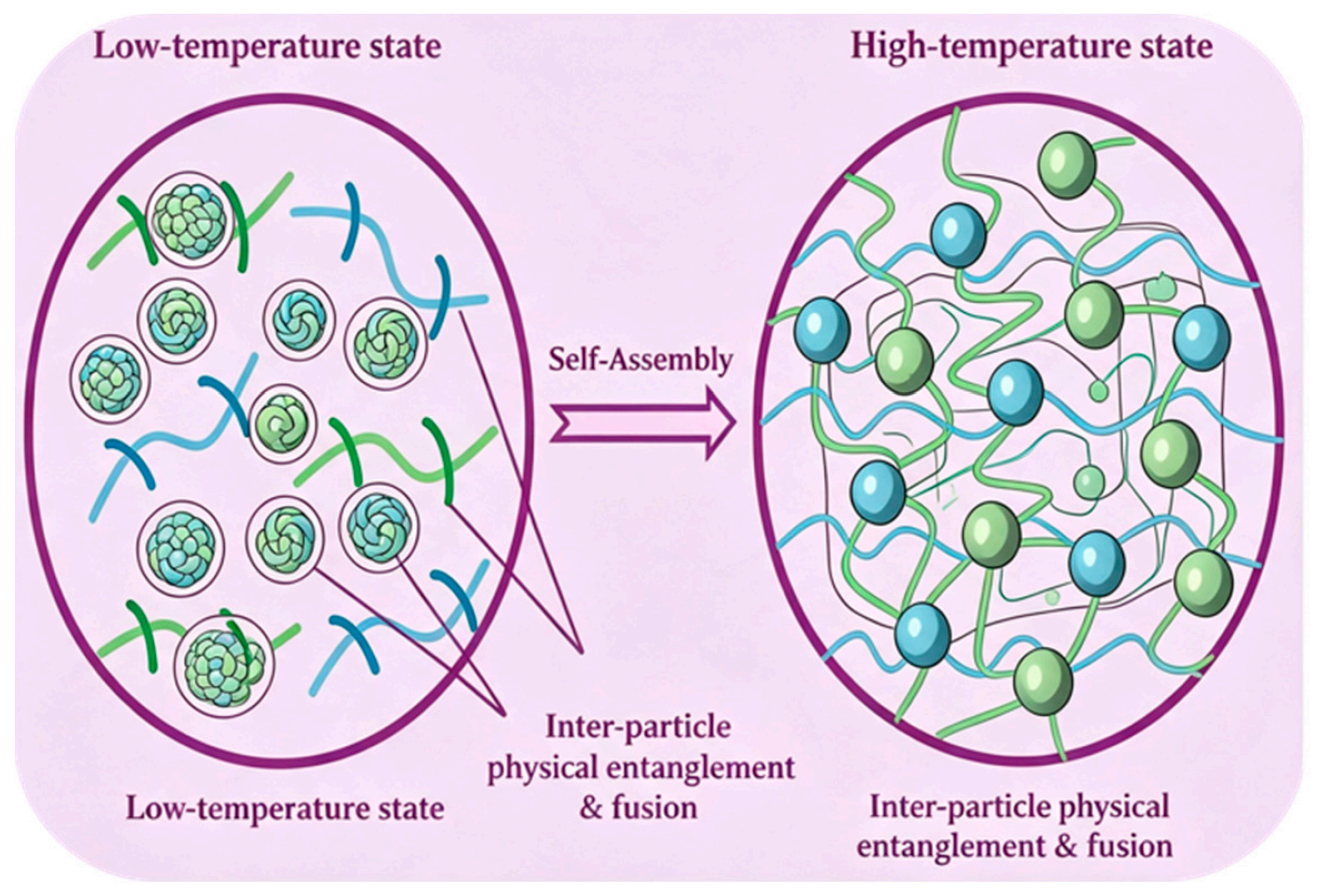
Schematic illustration of the thermo-responsive in situ gelation and plugging mechanism of SBR−g−PSA.

The thermo-responsive in situ gelation and plugging mechanism of SBR−g−PSA is schematically illustrated in Figure 14. The high-temperature sealing efficacy of SBR−g−PSA stems from a series of molecular conformational evolutions and physical entanglement interactions occurring within its unique graft copolymer network under thermal stimulation.

From a molecular structural design perspective, SBR−g−PSA is composed of a highly flexible polybutadiene main chain, a small number of rigid styrene microdomains providing physical crosslinking, and a high density of grafted octadecyl acrylate long side chains. In a low-temperature state, constrained by the strong binding forces of the physical crosslinking points, the grafted long side chains tightly coil around the core, resulting in a compact, shrunken particle with a small volume. As the temperature exceeds the crosslinking dissociation threshold (T > 80 °C), the binding forces within the macromolecular network significantly weaken. Driven by thermodynamics, the abundant octadecyl long side chains, due to their extreme lipophilicity, fully extend outward into the surrounding continuous oil phase. Interestingly, the macroscopic gelation kinetics exhibit a unique temperature dependence, as previously demonstrated by the gelation time extending from 100 min at 120 °C to 150 min at elevated temperatures (150–180 °C). This is governed by the competition between chain extension and thermal agitation. At a moderately high temperature (120 °C), the thermal energy is sufficient to activate chain unfolding and optimize the hydrophobic associations among the octadecyl chains, resulting in rapid gelation. However, as the temperature increases further (≥150 °C), the excessive kinetic energy induces intense Brownian motion of the polymer segments. This violent thermal agitation acts as a disruptive force against the non-covalent physical entanglements (van der Waals forces) [43]. Consequently, the polymer chains require a longer duration to establish a stable, percolating dynamic equilibrium against the thermal disruption, thus reasonably extending the macroscopic gelation time while ensuring a highly resilient final network.

As the long side chains fully unfold, the octadecyl chains of adjacent polymer molecules intensely penetrate and physically entangle with one another (primarily driven by strong hydrophobic associations and van der Waals forces). This novel network, formed by massive entanglement of lipophilic side chains, combined with the substantial influx of oil solvent, causes the gel particles to undergo significant oil-absorbing volumetric expansion. When the material is pumped into leakage fractures or pore throats, the previously independent dispersed microparticles instantaneously crosslink and fuse, self-assembling into a macroscopically continuous, dense, and elastic three-dimensional gel plug. Within this 3D network, the flexible polybutadiene backbone endows the gel with outstanding deformational adaptability and interfacial adhesion capabilities, while the high-density side-chain entanglements provide powerful elastic cohesive strength (macroscopically manifested as a storage modulus G′ vastly exceeding the loss modulus G″). When confronted with formation leakage channels, this in situ generated solid-like elastic gel firmly adheres to and is retained on oil-wet rock surfaces. Leveraging its high-modulus network, it effectively resists the dynamic washout of drilling fluids and high-pressure differentials, achieving an airtight seal with up to a 4 MPa pressure difference. This intelligent responsive mechanism of “temperature-triggered crosslinking relaxation–extension and entanglement of lipophilic side chains–in situ construction of 3D networks” fundamentally endows the material with its core capability for full-scale, long-term plugging [39]. While direct in situ microscopic imaging under combined 160 °C and 4 MPa conditions is constrained by current analytical apparatus, the simultaneous rise in high-temperature storage modulus (G′ ≫ G″) and the zero-fluid-loss fracture sealing performance jointly confirm the in situ macro-assembly of this dense physical network.

## 3. Field Application

Field application of SBR−g−PSA was conducted to address the high-temperature lost circulation conditions in Well Dashen-X in the Southwest Block. The well has a designed depth of 6338 m and a bottom-hole temperature of approximately 160 °C. OBDF was utilized while drilling the fifth hole section into the Dengying Formation. During drilling operations, severe lost circulation occurred multiple times, with an average loss rate reaching 10–15 m^3^/h and a cumulative loss of OBDF reaching 1800 m^3^. Conventional methods employed initially, such as bridging-while-drilling plugging, yielded unsatisfactory results.

To ensure the normal progress of subsequent drilling operations and to mitigate the costly fluid losses of the OBDF, the SBR−g−PSA thermo-responsive gel material developed in this study was selected for the plugging operation. Following simplification of the bottom hole assembly (BHA), a pre-mixed squeeze-plugging process was implemented. The specific operational design was as follows:

(1) Wellbore Profile and BHA

The wellbore profile and BHA configuration are summarized in Table 1.

(2) Drilling Fluid System and Properties

An OBDF system was utilized with the following parameters: density 1.45 g/cm^3^, funnel viscosity 65 s, apparent viscosity 44 mPa·s, plastic viscosity 36 mPa·s, yield point 8 Pa, initial/final gel strength 3/8 Pa, alkalinity 3.5, sand content 0.3%, solid phase content 20%, oil/water ratio 80:20, HTHP fluid loss (at 130 °C) 2.2 mL, Cl^−^ concentration 27,000 mg/L, Ca^2+^ concentration 12,000 mg/L, and electrical stability 635 V.

(3) Plugging Fluid Formulation

The base drilling fluid formulation was + 2% JD-5 + 4% GT-MF + 2% NTBASE + 2% LCM-3 + 3% LCM-2 + 4% LCM-1 + 2% WNDK-3 + 3% WNDK-4 + 3% SBR−g−PSA + 6% ultrafine calcium carbonate + 3% HR-2 Type 1 + 3% WNSOL. The effective pumping volume was 20 m^3^.

(4) Operational Procedures

Precise managed pressure circulation indicated an average leakage rate of 2.42 m^3^/h, with 8 m^3^ of OBDF (density 1.45 g/cm^3^) lost.

The drill string was tripped with back-reaming to a depth of 6256.00 m, and the fluid level was observed to be normal. 70.0 m^3^ of OBDF (density 1.45 g/cm^3^) was forward-pumped to ensure all plugging fluid exited the bit nozzles, during which 7.6 m^3^ of OBDF was lost.

The drill string was securely pulled to a depth of 5346.62 m. The semi-blind blowout preventer was closed to hold pressure and wait for the plug to set. Through an intermittent forward squeezing process, a cumulative 13.8 m^3^ of the plugging fluid was squeezed into the formation (equating to the lost volume of the plug slug).

The string was run back down to a depth of 5973.00 m, maintaining a normal fluid level. Carefully managed pressure circulation showed no noticeable drop in the fluid level. Subsequently, the pumps were engaged to ream down to a depth of 6144.00 m, with the fluid level remaining consistently stable.

During field execution, the total duration for mixing the 20 m^3^ plugging slurry and displacing it to the target depth of 6256 m was approximately 110 min. Premature gelation within the drill string was effectively prevented by two factors: first, continuous drilling fluid circulation maintained the string’s internal temperature well below the bottom-hole static temperature (160 °C); second, the intense dynamic shear forces exerted inside the drill pipe disrupted early physical association nodes, ensuring that macro-gelation occurred only after the slug entered the high-temperature, low-shear environment of the formation fractures.

(5) Application Performance Evaluation

The high-temperature-resistant (180 °C) oil-based expansive plugging technology was successfully implemented in Well Dashen-X of the Southwest Block. By scientifically incorporating the independently developed SBR−g−PSA into the composite formulation, a strong synergistic effect was achieved between the thermo-responsive gel and traditional rigid plugging materials (e.g., ultrafine calcium carbonate) [44]. While the rigid particles bridged the main fracture apertures, SBR−g−PSA expanded in situ under high temperatures to tightly seal the remaining micro-voids, collectively reducing the formation leakage rate from 2.42 m^3^/h to 0 m^3^/h. Field engineering practice definitively proves that this intelligent responsive plugging technology can effectively solve the severe leakage challenges currently faced by deep-well OBDFs in high-temperature fractured intervals.

## 4. Conclusions

Guided by macromolecular architecture design, a thermo-responsive gel plugging material (SBR−g−PSA) was successfully synthesized and applied for oil-based lost circulation control. The major conclusions and universal insights are as follows:

(1) Entropy-Driven Smart Responsiveness: The incorporation of lipophilic C18 side chains onto a flexible SBR backbone achieves a UCST-like phase transition. Dissociation of physical crosslinks above 80 °C triggers in situ side-chain extension and physical entanglement, constructing a robust 3D network at downhole temperatures (120–180 °C).
(2) High Thermal & Mechanical Resilience: SBR−g−PSA exhibits excellent thermal stability up to 346.6 °C. The gel network maintains strong elastic characteristics (G′ ≫ G″) up to 160 °C, enabling complete pressure-bearing sealing (≥4″ MPa”) across 1–1.5 mm fractures and sand beds.
(3) Balanced Drilling Fluid Compatibility: SBR−g−PSA seamlessly disperses in OBDFs without destabilizing electrical stability or rheology at shallow depths, while offering a controllable gelation time window (100–150 min) suitable for safe pumping operations.
(4) Field Validation: Successful deployment in Well Dashen-X completely eliminated severe leakage (reducing loss rate from 2.42 m^3^/h to 0 m^3^/h), establishing a viable paradigm for deep-well lost circulation control.

Future Work: Future research will concentrate on systematically mapping the responsive kinetics under ultra-high salinity and multi-phase shear fields, alongside evaluating the long-term environmental degradation pathways of lipophilic synthetic polymers for sustainable drilling operations.

## 5. Materials and Methods

### 5.1. Materials and Reagents

Styrene–butadiene rubber (SBR) was purchased from Shanghai Jizhi Biochemical Technology Co., Ltd. (Shanghai, China). Stearyl acrylate (SA) and benzoyl peroxide (BPO) were procured from Shanghai Macklin Biochemical Technology Co., Ltd. (Shanghai, China). All chemical reagents utilized were of analytical purity grade and were used directly without any additional purification. The solvent toluene and precipitant anhydrous ethanol used in the experiments were also of analytical grade and were supplied by Shanghai Aladdin Biochemical Technology Co., Ltd. (Shanghai, China). Deionized water was utilized throughout the entirety of the experimental procedures.

Diesel oil (density: 0.83 g/cm^3^, kinematic viscosity at 40 °C: 3.2 mm^2^/s) was supplied by Sinopec Co., Ltd. (Beijing, China). Organoclay, oxidized lime (CaO), and lipophilic emulsifiers were industrial-grade additives provided by Chuanqing Drilling Engineering Co., Ltd. (Chengdu, China).

### 5.2. Synthesis of SBR−g−PSA

In this study, a solution free-radical graft copolymerization method was employed to synthesize the oil-based thermo-responsive gel base agent [45]. Specifically, the highly lipophilic long-chain monomer stearyl acrylate (SA) was grafted onto the styrene–butadiene rubber (SBR) backbone under the hydrogen-abstraction action of an initiator. The detailed synthetic procedure is as follows: First, 200 mL of toluene solvent was added to a 500 mL three-neck round-bottom flask equipped with a mechanical stirrer, a reflux condenser, and a thermometer. 5.0 g of chopped SBR rubber pieces was subsequently added and continuously stirred at room temperature for 4 h until complete dissolution, yielding a homogeneous polymer solution. Following this, high-purity nitrogen gas was bubbled through the flask for 30 min to thoroughly eliminate oxygen from the reaction system. The water bath temperature was then raised and maintained at 85 °C. To prevent the uncontrollable macroscopic bulk crosslinking of the polybutadiene double bonds during the free-radical polymerization, the reaction was strictly maintained under highly dilute solution conditions (SBR concentration < 5 wt%). Additionally, the reaction kinetics were controlled by optimizing the specific low dosage of the BPO initiator. Specifically, 15.0 g of SA monomer and 0.15 g of the hydrogen-abstracting initiator BPO were dissolved in a small amount of toluene and added dropwise into the reaction flask over 1.5 h using a constant-pressure dropping funnel. Upon completion of the dropwise addition, the reaction was allowed to proceed under continuous mechanical stirring at a constant temperature of 85 °C under nitrogen protection for 6 to 8 h. After the reaction concluded, the system was allowed to naturally cool to room temperature. The viscous reaction mixture was then slowly poured into a large volume of vigorously stirred anhydrous ethanol to precipitate the product. Once the graft copolymer was fully precipitated, the solid was collected via vacuum filtration and subsequently subjected to Soxhlet extraction using anhydrous ethanol for 12 h to completely remove any unreacted SA monomer and its homopolymers. Finally, the purified product was dried to a constant weight in a vacuum drying oven at 60 °C, obtaining the target graft copolymer (SBR−g−PSA). The Grafting Yield (GY) and Grafting Efficiency (GE) of the purified product were calculated to be 68.4% and 72.1%, respectively, using the following equations:

$$GY\left(\%\right)=\frac{W_{g}-W_{0}}{W_{0}}\times 100\%$$

$$GE\left(\%\right)=\frac{W_{g}-W_{0}}{W_{m}}\times 100\%$$

where $W_{0}$ is the initial weight of SBR (5.0 g), $W_{m}$ is the weight of the SA monomer (15.0 g), and $W_{g}$ is the final weight of the purified grafted product.

### 5.3. Characterization

#### 5.3.1. FTIR Characterization

The molecular structure of the synthesized SBR−g−PSA was analyzed using a Fourier-Transform Infrared (FTIR) spectrometer (Thermo Fisher Nicolet iS50, Waltham, MA, USA) with the KBr pellet technique and 32 scans from 4000 to 400 cm^−1^ with a resolution of 4 cm^−1^ to confirm the successful grafting and the incorporation of characteristic functional groups.

#### 5.3.2. ^1^H–NMR Characterization

To further elucidate the chemical composition and aggregation morphology, solid-state 1H-NMR spectroscopy was performed on the purified solid graft copolymer using a Bruker Avance III 400 MHz solid-state NMR spectrometer (Billerica, MA, USA) equipped with a 4 mm MAS probe at a spinning rate of 10 kHz. The spectra were analyzed to evaluate the segmental motion characteristics and confirm the successful grafting of the high-density octadecyl long chains onto the polymer backbone.

#### 5.3.3. Micro−Morphology Characterization

The micro-morphology and microphase aggregation patterns of the SBR−g−PSA samples were investigated using optical microscopy (Carl Zeiss, Oberkochen, Germany). This allowed for the direct observation of the gel’s dispersion state and the distinct structural distribution of the lipophilic aggregation core and the flexible polymer network skeleton.

#### 5.3.4. Particle Size Characterization 

The particle size distribution of the SBR−g−PSA microgel was measured using a Malvern Mastersizer 3000 laser diffraction particle size analyzer (Malvern, Worcestershire, UK) with diesel as the dispersant. The median particle diameter (D50) was recorded to verify the size adaptability of the micro/nanoscale particles and their physical foundation for full-scale plugging.

#### 5.3.5. Thermogravimetric Analysis (TGA)

The thermal stability and degradation characteristics of the gel were evaluated via TGA using a TA Instruments Q500 analyzer (New Castle, DE, USA), heated from ambient temperature to 600 °C at a heating rate of 10 °C/min under a continuous N_2_ flow (50 mL/min). The samples were heated up to 600 °C, and the resulting TGA and derivative thermogravimetry (DTG) curves were recorded to determine the onset thermal degradation temperature (T_5%_), maximum thermal decomposition rate temperature (T_max_), and final char yield.

### 5.4. Fluid Preparation

All performance evaluations were conducted utilizing a standardized base oil-based drilling fluid (OBDF). The base formulation consisted of diesel, 6% emulsifier, 3% organoclay, 3% calcium oxide, and 30 mL of brine (25% mass fraction). Barite was added to adjust the final fluid density to 1.5 g/cm^3^. The SBR−g−PSA plugging agent was subsequently added to the base fluid at designated mass fractions (e.g., 5% and 10%) and stirred uniformly for subsequent rheological and plugging tests.

### 5.5. Rheological and Mechanical Measurements

#### 5.5.1. Rheological Properties and Gelation Time

The rheological parameters of the OBDFs, including apparent viscosity, yield point, high-temperature–high-pressure (HTHP) fluid loss, and electrical stability, were measured before and after dynamic aging for 16 h at 160 °C. Additionally, the gelation time of the SBR−g−PSA system was systematically recorded at specific target temperatures ranging from 120 °C to 180 °C to evaluate its thermal responsiveness and controllability.

#### 5.5.2. Measurement of Adhesion and Retention

To assess the retention performance on rock surfaces, micro-mechanical evaluations were conducted using an atomic force microscope. The adhesive force between a SiO_2_ microsphere probe and the OBDFs containing SBR−g−PSA was measured. Testing variables included the applied force of the probe (100–1000 nN), residence time (0.1–5.0 s), and relative penetration speed (3–78.1 μm/s) to evaluate the fluid’s resistance to dynamic washout.

#### 5.5.3. Measurement of Dynamic Viscoelasticity and Gel Strength

The mechanical strength of the gel was evaluated using dynamic oscillatory shear sweeps after 72 h of high-temperature aging at 120 °C, 140 °C, and 160 °C. The storage modulus (G′) and loss modulus (G″) were continuously recorded across a range of shear stresses to characterize the solid-like elastic nature, structural integrity, and pressure-bearing resilience of the polymer network.

### 5.6. Lost Circulation Performance Testing

#### 5.6.1. Fracture Sealing Test

The sealing efficacy in fractured formations was evaluated using an HTHP lost circulation testing apparatus equipped with 1 mm and 1.5 mm slotted metal plates [46]. The test fluid (OBDF containing 10% SBR−g−PSA) was pressurized under a stepwise gradient up to a maximum differential pressure of 4 MPa within a temperature range of 120–160 °C. The cumulative fluid loss was recorded to verify the pressure-bearing capacity of the formed sealing layer.

#### 5.6.2. Sand Bed Plugging Test

To evaluate the plugging performance in highly permeable formations, dynamic filtration tests were conducted using the HTHP tester filled with a 20–40 mesh quartz sand bed. The SBR−g−PSA-enhanced OBDF was subjected to systematic pressurization, heating (120 °C, 140 °C, and 160 °C), and stabilization procedures. The cumulative fluid loss at up to 4 MPa differential pressure was measured to determine the temperature-dependent compressive resistance and overall leak-prevention efficacy of the gel network.

During the preparation of this manuscript, generative artificial intelligence (Gemini) was utilized strictly for English language polishing and grammatical editing to improve readability. No AI tools were used in data generation, physical testing, or the synthesis and evaluation of the SBR−g−PSA gel.

## Figures and Tables

**Table 1:** Wellbore profile and bottom hole assembly (BHA) for the plugging operation in Well Dashen-X.

Casing Name	Depth (m)	Formation	Hole Size (mm)
Surface Casing	300	Suining Fm.	660.4
Technical Casing 1	2856	Leikoupo Fm.	455
Technical Casing 2	5306	Emeishan Basalt	333.4
Production Liner	5950	Dengying Fm.	241.3
Production Tie-back	5950	Dengying Fm.	241.3
Open Hole	6338	Dengying Fm.	160

## Data Availability

Data are contained within the article.
